# Susceptibility of cyclin-dependent kinase inhibitor 1-deficient mice to rheumatoid arthritis arising from interleukin-1β-induced inflammation

**DOI:** 10.1038/s41598-021-92055-9

**Published:** 2021-06-15

**Authors:** Yoshinori Takashima, Shinya Hayashi, Koji Fukuda, Toshihisa Maeda, Masanori Tsubosaka, Tomoyuki Kamenaga, Kenichi Kikuchi, Masahiro Fujita, Yuichi Kuroda, Shingo Hashimoto, Naoki Nakano, Tomoyuki Matsumoto, Ryosuke Kuroda

**Affiliations:** Department of Orthopaedic Surgery, Kobe University Graduate School of Medicine, 7-5-2, Kusunoki-chou, Chuo-ku, Kobe, Hyogo 650-0017 Japan

**Keywords:** Acute inflammatory arthritis, Rheumatoid arthritis

## Abstract

We recently reported that cyclin-dependent kinase inhibitor 1 (p21) deficiency induces osteoarthritis susceptibility. Here, we determined the mechanism underlying the effect of p21 in synovial and cartilage tissues in RA. The knee joints of p21-knockout (p21^−/−^) (*n* = 16) and wild type C57BL/6 (p21^+/+^) mice (*n* = 16) served as in vivo models of collagen antibody-induced arthritis (CAIA). Arthritis severity was evaluated by immunological and histological analyses. The response of p21 small-interfering RNA (siRNA)-treated human RA FLSs (*n* = 5 per group) to interleukin (IL)-1β stimulation was determined in vitro. Arthritis scores were higher in p21^−/−^ mice than in p21^+/+^ mice. More severe synovitis, earlier loss of Safranin-O staining, and cartilage destruction were observed in p21^−/−^ mice compared to p21^+/+^ mice. p21^−/−^ mice expressed higher levels of IL-1β, TNF-α, F4/80, CD86, p-IKKα/β, and matrix metalloproteinases (MMPs) in cartilage and synovial tissues via IL-1β-induced NF-kB signaling. IL-1β stimulation significantly increased IL-6, IL-8, and MMP expression, and enhanced IKKα/β and IκBα phosphorylation in human FLSs. p21-deficient CAIA mice are susceptible to RA phenotype alterations, including joint cartilage destruction and severe synovitis. Therefore, p21 may have a regulatory role in inflammatory cytokine production including IL-1β, IL-6, and TNF-α.

## Introduction

Rheumatoid arthritis (RA) is characterized by chronic synovial inflammation of multiple joints^1,2^. The affected synovial tissues contain activated macrophages, fibroblasts, and T and B lymphocytes induced by pro-inflammatory cytokines, such as interleukin (IL)-1β, tumor necrosis factor-α (TNF-α), and IL-6. The cytokines provoke and perpetuate the synovial membrane inflammation in the joints, which leads to articular cartilage damage and bone erosion^3,4^. Especially, activated RA fibroblast-like synoviocytes (FLSs) contribute to the inflammatory and destructive potential of the aggressive pannus tissue in patients with RA by producing pro-inflammatory mediators and matrix metalloproteinases (MMPs), such as MMP-1, MMP-3, and MMP-9^5–11^. Moreover, IL-1β is produced by chondrocytes, osteoblasts, mononuclear cells, and cells forming synovial membranes and secreted into the hip and knee joints during an inflammatory response^12^. IL-1β induces the synthesis of MMPs, specifically MMP-13, in the chondrocytes, resulting in cartilage destruction^13^. Patients with RA display elevated IL-1β levels in FLSs, the synovial fluid, the synovial membrane, cartilage, and the subchondral bone layer^6^, suggesting that IL-1β is involved in the pathogenesis and progression of RA.

Animal models of RA, including rat and mouse models of type II collagen-induced arthritis, rat models of adjuvant-induced arthritis, and antigen-induced arthritis models in several species, have proven to be highly predictive of therapeutic efficacy in humans^14^. In the mouse model of collagen antibody-induced arthritis (CAIA), severe arthritis is induced within 24–48 h and reaches its peaked on day 7–8, whereas bone degradation progresses until day 21^15^. In particular, arthritis is induced through systemic administration of antibody mixtures that target various type II collagen epitopes. Therefore, this model is ideal for rapid screening of novel arthritis therapeutics and elucidating the mechanisms underlying arthritis development^16^. Moreover, this method can induce arthritis in various mouse strains, not just CAIA-susceptible mice, making it ideal for studying the pathological role of individual gene products, such as cytokines, without the influence of complete or incomplete Freund’s adjuvant that may strongly affect the host immune system.

Cyclin-dependent kinase (CDK) inhibitor 1 (p21) was initially identified as a potent inhibitor of cell-cycle progression^17–20^. The knockdown of p21 induces a regenerative response in an appendage of an otherwise non-regenerating mouse strain^21^. It has been shown to regulates cell proliferation and inflammation after arterial injury in local vascular cells^22^. Subsequent studies also confirmed that p21 induces resistance to apoptosis, and regulated inflammation, cytostasis, and cell death^23^. p21^−/−^ mice have been shown to lack any overt skeletal phenotype and are unlikely to develop spontaneous malignancies^24–26^.

We recently reported that p21 deficiency induces susceptibility to osteoarthritis (OA) through signal transducer and activator of transcription 3 (STAT3)- and IL-1β-induced activation of nuclear factor kappa-light-chain-enhancer of activated B cells (NF-κB) signaling^27,28^. NF-κB is activated by inflammatory cytokines, including IL-1β and TNF-α, and promotes macrophage relocalization. Macrophage cells differentiate from monocytes following exposure to a specific cytokine milieu. Activated macrophages, especially M1 macrophages, produce IL-1β, IL-6, and TNF-α, among other pro-inflammatory cytokines, thereby enhancing systemic and local inflammation^29,30^. Recent studies have also shown that p21 plays a key role in suppressing activated macrophages^31^, and that expression of p21 in rheumatoid synovial fibroblasts resulted in downregulation of inflammatory mediators and tissue-degrading proteinases in RA^32^. While the expression of *p21* is decreased in RA FLSs^33^, it is unclear how p21 affects the joint synovial tissues and cartilage in RA.

In this study, we hypothesized that p21 deficiency enhances joint inflammation and cartilage destruction in RA. We evaluated the degree of joint inflammation to determine the mechanisms associated with p21 function in vitro and in vivo using RA FLS and the systemic arthritis model, respectively.

## Methods

### Generation of homozygous p21^−/−^ mice

Homozygous B6.129S6 (Cg)-Cdkn1atm1 Led/J mice were obtained from the Jackson Laboratory (Bar Harbor, ME, USA). We backcrossed these mice for 10 generations against a C57BL/6 background, obtained from CREA Japan, Inc (Tokyo, Japan), and studied 10-week-old male mice (*n* = 16). p21^+/+^ littermates were used as wild type (WT) controls (*n* = 16). Genotyping was performed using polymerase chain reaction (PCR)-based amplification of mouse-tail DNA with allele-specific probes. Both the p21^+/+^ and p21^−/−^ groups contained four mice. All animals were bred in mouse houses with automatically controlled lighting (12 h light/dark cycle) and a stable temperature of 23 °C and were allowed ad libitum access to food and water throughout the study. This study was performed in strict accordance with the recommendations of the Guide for the Care and Use of Laboratory Animals published by the National Institutes of Health (Bethesda, MD, USA). All procedures were approved by the Animal Studies Committee of Kobe University, Japan (permit number: P180404). Confirms that the authors complied with the ARRIVE guidelines.

### Establishment of a CAIA mouse model

A cocktail of five monoclonal antibodies recognizing conserved epitopes on various species of type II collagen (Chondrex Inc., Redmond, WA, USA) was prepared as previously described and used according to the manufacturer’s instructions. Mice were injected with the cocktail of antibodies intraperitoneally (i.p.; 5 mg). Three days later, they were injected with 50-µg lipopolysaccharide (LPS) from *Escherichia coli* 0111: B4 (Chondrex Inc.) i.p. to induce arthritis. On days 7, 14, and 28 (counting from day 0 as the day of cocktail injection), at least four mice each from the p21^−/−^ and WT groups were euthanized using CO_2_. We defined the mice without injection of monoclonal antibodies as the control mice.

### Evaluation of arthritis

The mice were blindly evaluated for disease progression on days 0 (*n* = 32), 3 (*n* = 24), 7 (*n* = 24), 10 (*n* = 16), 14 (*n *= 16), and 28 (*n* = 8). The severity of arthritis in each joint was graded macroscopically on a scale of 0–4, as follows: 0, normal; 1, mild swelling; 2, moderate swelling; 3, severe swelling; 4, pronounced edema of the entire paw by triple-blinded observers. The cumulative score from all four paws (maximum score of 16 per mouse) was used as the overall disease score^34^.

### Histological evaluation for cartilage degeneration and synovitis

Mouse knee joints were fixed using 4% paraformaldehyde (Wako, Osaka, Japan) for 24 h, decalcified with 14% ethylenediaminetetraacetic acid (EDTA; Dojindo, Kumamoto, Japan) for 7 days, and embedded in paraffin. Histological coronal sections were obtained from the joint at 80-µm intervals and stained with Safranin-O (Tokyo Chemical Industry, Tokyo, Japan) and Fast Green (Chroma-Gesellschaft, Thermo Fisher Scientific, Inc). RA histopathology was evaluated using the Osteoarthritis Research Society International (OARSI) cartilage OA-histopathology scoring system. Histological scores were measured in the four quadrants (i.e., medial femoral condyle, medial tibial plateau, lateral femoral condyle, and lateral tibial plateau) of the knee joints at all sectioned levels (eight sections per knee) to obtain summed scores. The summed scores were calculated from all four quadrants of all sections that represented whole-joint changes^35^. Synovitis was also evaluated using the OARSI-recommended scoring system of hematoxylin (Muto Pure Chemicals, Tokyo, Japan) and eosin (Fujifilm, Tokyo, Japan) stained section^36^. Two specimens from each compartment were evaluated, and the highest score was recorded. The average of each compartment score was considered as the whole-knee score. Coronal sections from the 16 mice were evaluated for each group.

### Enzyme-linked immunosorbent assay (ELISA)

Serum levels of IL-1β, IL-6, and TNF-α (*n* = 32) were detected using a sandwich ELISA kit according to the manufacturer’s instructions (Abcam, Cambridge, UK). ELISA results were quantitated by measuring the absorbance at 450 nm on a microplate reader (Bio-Rad, Hercules, CA, USA) and normalized by the number of cells per well.

### Immunohistochemistry (IHC)

Deparaffinized sections were digested with proteinase (Dako, Glostrup, Denmark) for 10 min and treated with 3% hydrogen peroxide (Wako, Osaka, Japan) to block endogenous peroxidase activity. We assessed F4/80 expression—using a previously reported scoring system for immunohistochemistry—as an immune and inflammatory cell marker because it is a well-known macrophage marker^37^, and to investigate the M1/M2 ratio, we assessed CD86 and CD206 expression as M1 and M2 macrophage markers, respectively^38^.

The sections were probed with the following antibodies (1:50 dilution) at 4 °C overnight: anti-F4/80 (AbDSerotec, Kidlington, UK), anti-CD86 (Abcam, Cambridge, UK), anti-CD206 (Abcam, Cambridge, UK), anti-IL-1β (Abcam, Cambridge, UK), anti-TNF-α (GeneTex, Irvine, CA, USA), phospho-IκB kinase complex (IKK) α/β (Cell Signaling Technology, Danvers, MA, USA), anti-MMP-3 (Santa Cruz Biotechnology, Dallas, TX, USA), anti-MMP-13 (Abcam, Cambridge, UK), or anti-MMP-9 (Proteintech Group, Chicago, IL, USA). Sections were subsequently probed with peroxidase-labeled anti-rabbit or anti-rat IgG (Histofine Simple Stain MAX PO; Nichirei Bioscience, Tokyo, Japan) antibody at 23–27 °C for 1 h. The brown reaction product was generated as a signal upon addition of the peroxidase substrate 3,3′-diaminobenzidine for 5 min (Histofine Simple Stain DAB solution; Nichirei Bioscience, Tokyo, Japan), and the sections were examined under an optical microscope. Hematoxylin was used as a counterstain.

One coronal section from the center of the most severe lesion in each tibial plateau was scored. The numbers of stained cells were counted in three areas of high-magnification fields at both superficial and deep zones of the cartilage tissue by triple-blinded observers. The average percentages of MMP-3-, MMP-13-, p-IKKα/β-, TNF-α-, IL-1β-positive cells relative to total hematoxylin-stained cells were calculated. The positive cells were included superior of the tidemark. CD86 and CD206 expression levels in the synovium were determined semi-quantitatively using the National Institutes of Health ImageJ software (http://imagej.nih.gov/ij/) and digitally captured images. Expression levels were determined as the average of the gray values normalized by the number of nuclei, similar to a previously published method^39^. In brief, for color deconvolution of IHC images, DAB and hematoxylin staining were digitally separated using ImageJ software with a color deconvolution plugin. Deconvoluted images with DAB staining were subjected to measurement of mean gray values, with the lower and upper thresholds set at 0 and 120 for CD86 and CD206, respectively. Coronal sections from the 16 mice were evaluated for each group.

### Preparation of human synovium

Synovial tissues were obtained during a total knee joint replacement surgery from five patients with RA. All RA patients fulfilled the American College of Rheumatology 1987 revised criteria for RA^40^. OA synovial tissues were also obtained during total knee joint replacement surgery from five patients as controls. Diagnoses of OA were based on clinical, laboratory, and radiographic evaluations. All samples were obtained following the World Medical Association Declaration of Helsinki Ethical Principles for Medical Research Involving Human Subjects. The study protocol was approved by the Kobe University Graduate School of Medicine Ethics Committee, and all participants provided informed consent.

### Preparation of cell culture

Primary synoviocytes were isolated and cultured from the RA and OA synovial tissues. The tissues were minced and incubated with trypsin (0.5 mg/ml; Sigma-Aldrich, St. Louis, MO, USA) for 15 min at 37 °C, and then the synovium was treated with Dulbecco’s modified Eagle’s medium (DMEM; Gibco/Life Technologies, Grand Island, NY, USA) containing 0.2% collagenase (Sigma-Aldrich, St. Louis, MO, USA) at 37 °C for 15 h. Dissociated cells were cultured overnight in DMEM supplemented with 10% fetal bovine serum (BioWhittaker FBS; Lonza, Walkersville, MD, USA) and 100 U/ml penicillin–streptomycin. The non-adherent cells were removed, and the adherent cells were further incubated on a 6-well plate with fresh medium (3 × 10^5^ cells/well). All experiments were conducted using 3–5 passage cells.

### Transfection of small-interfering RNA (siRNA)

Lipofectamine RNAiMax transfection reagent (Invitrogen, Carlsbad, CA, USA) was used to transfect *p21* siRNA and nonspecific siRNA control into the RA and OA human knee synoviocytes, respectively, according to the manufacturer’s recommendations. Briefly, a day before transfection, the cells (3 × 10^5^ cells/well) were seeded on a 6-well plate without antibiotics to achieve 30–50% confluence at the time of transfection. Subsequently, 5 pmol of siRNA and Lipofectamine RNAiMax complexes were prepared and added to each well. After transfection for 24 h, the complexes were removed, and fresh medium containing 10% FBS was added.

### Quantitative reverse transcriptase–polymerase chain reaction (RT–PCR)

Cultured RA and OA synoviocytes were transfected with the p21 siRNA or nonspecific siRNA control. FLSs without siRNA transfection were used as controls. After transfection for 24 h, the cells were incubated for another 24 h with or without stimulation with 10-ng/ml recombinant human IL-1β (R&D systems, McKinnley, MN, USA), followed by RNA extraction using a QIA shredder and RNeasy Mini Kit (Qiagen, Hilden, Germany) according to the manufacturer’s protocol. Briefly, 1 μg of total RNA was reverse-transcribed to first-strand cDNA using 1.25-μM oligo-dT primer in 40-μl PCR buffer II containing 2.5-mM MgC1_2_, 0.5-mM dNTP mix, 0.5 U of RNase inhibitor, and 1.25 U of murine leukemia virus reverse transcriptase (PerkinElmer/Applied Biosystems, Foster City, CA, USA), at 42 °C for 1 h. The relative expression levels of mRNA of human *p21*, *IL-6*, *IL-8*, *MMP-3*, and *MMP-9*, were analyzed using SYBR Green RT–PCR on an ABI Prism 7500 sequence detection system (Applied Biosystems, Foster City, CA, USA). Relative gene expression was normalized against the Glyceraldehyde 3-phosphate dehydrogenase (GAPDH) housekeeping gene using the comparative cycle threshold (Ct) method. The difference between the mean Ct values of the gene of interest and those of the housekeeping gene is denoted as ΔCt, whereas the difference between the ΔCt and the Ct value of the calibrator sample is denoted as ΔΔCt. The log_2_ (ΔΔCt) value gives the relative level of gene expression. The primer sequences used to detect human *p21*, *IL-6*, *IL-8*, *MMP-3*, and *MMP-9*, are listed in Supplementary Table S1.

### Western blot analysis

First, the cultured RA synoviocytes were treated with or without 10-ng/ml recombinant human IL-1β (R&D Systems, Minneapolis, Minnesota, USA) for 5, 10, 15, 30, 60 min, and 24 h; stimulation time for IL-1β was determined as previously reported^41^. The synoviocytes were washed with Tris-buffered saline with Tween-20 (TBST) and lysed in a buffer containing 25-mM Tris, 1% Nonidet P-40, 150 mM NaCl, 1.5 mM ethylene glycol tetraacetic acid, and a protease/phosphatase inhibitor mix (Roche Diagnostics, Basel, Switzerland). The lysates were centrifuged at 4 °C at 15,000×*g* for 10 min to remove cellular debris. Next, the cellular debris-free lysates were collected and mixed with 4× electrophoresis sample buffer; 15 μl of cell lysates (1.0 × 10^7^ cells/ml) were electrophoresed on a 7.5–15% SDS‑polyacrylamide gradient gel (Biocraft, Tokyo, Japan) and electrically transferred onto a polyvinylidene difluoride blotting membrane (GE Healthcare Life Sciences, Little Chalfont, UK). The membrane was blocked with 5% skimmed milk in TBST at 25 °C for 30 min, incubated with antibodies against anti-p-IKKα/β (Cell Signaling Technology, Danvers, MA, USA), anti- phosphor-inhibitor of κB (IκB) α (Abcam, Cambridge, UK) and anti-IκBα (Abcam, Cambridge, UK) at 4 °C for 12 h, and further incubated with horseradish peroxidase-conjugated goat anti-rabbit IgG secondary antibody at 25 °C for 1 h. The proteins were subsequently visualized using ECL Plus reagent (GE Healthcare Life Sciences, Little Chalfont, UK) in a chemilumino analyzer (LAS-3000 mini; Fujifilm, Tokyo, Japan).

The cultured RA and OA synoviocytes were then transfected with p21 siRNA or nonspecific siRNA control. After 24 h of transfection, the cells were incubated with or without IL-1β stimulation for the period with the highest level of p-IKKα/β, p-IκBα, and IκBα in the western blot. Western blots of the synoviocytes were subsequently subjected to the same procedure as described above. Expression of the alpha-tubulin protein was detected using rabbit anti-alpha-tubulin polyclonal antibody (Abcam, Cambridge, UK) as a primary antibody. Protein expression was determined semi-quantitatively with the National Institutes of Health ImageJ using digitally captured images. Five different samples were analyzed for each experiment.

### Statistical analysis

**Figure 1:**
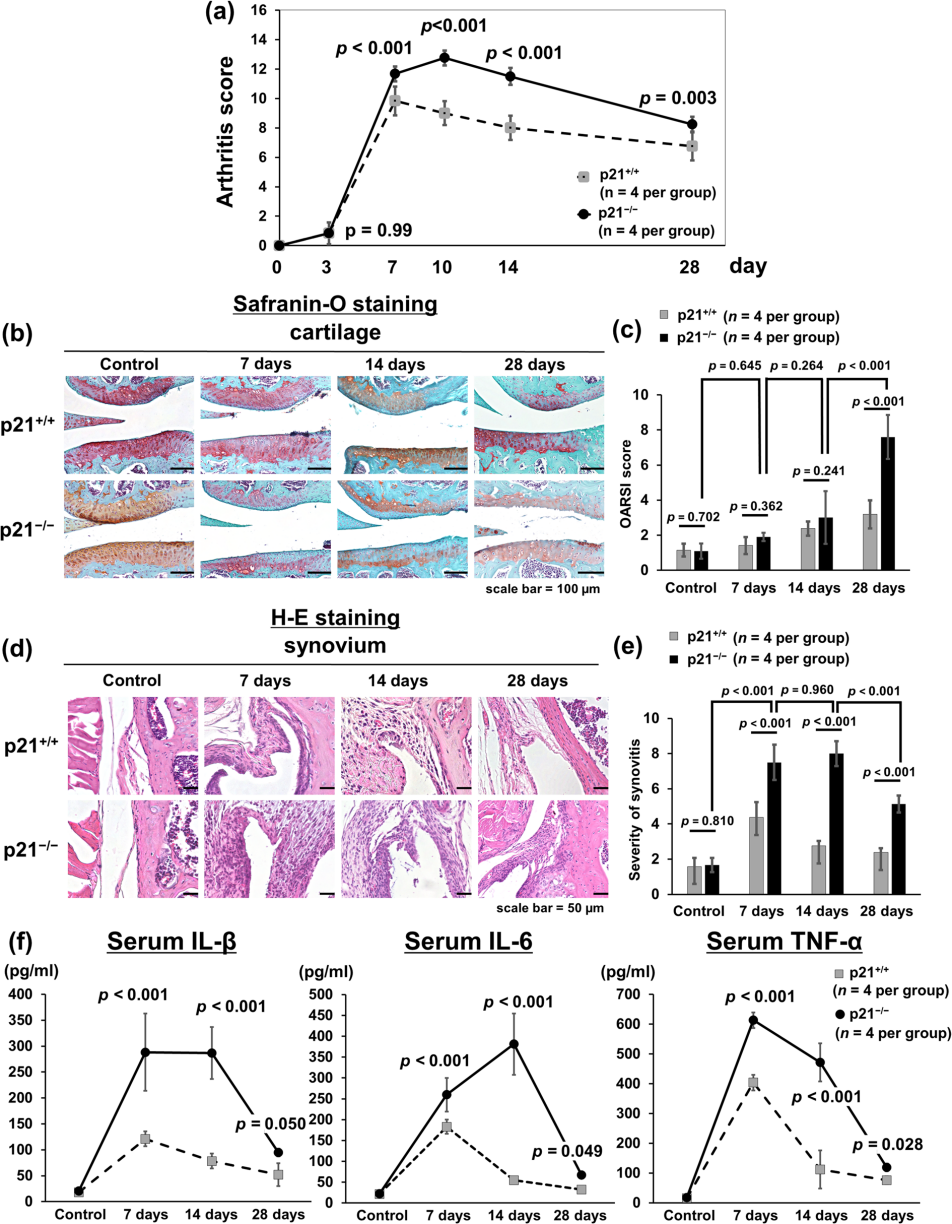
p21-deficient mice exhibit destructed knee joint, increased inflammatory arthritis, and enhanced systemic inflammatory cytokine expression in vivo. (**a**) OARSI-based arthritis scores of p21^−/−^ and p21^+/+^ mice on days 7, 10, 14, and 28 after administration of the antibody cocktail. Cartilage and synovial tissue samples were collected from the knee and stained with (**b**) Safranin-O and Fast Green, and (**d**) hematoxylin and eosin, respectively. (**b,d**) p21^+/+^ mice and p21^−/−^ mice as controls and on days 7, 14, and 28. (**c**) Average sum of the OARSI cartilage OA-histopathology scores and (**e**) average severity of synovitis scores with 95% CI from four quadrants (i.e., medial femoral condyle, medial tibial plateau, lateral femoral condyle, and lateral tibial plateau) and eight sections per knee. Four mice were analyzed from each group. (**f**) Serum levels of IL-1β, IL-6 and TNF-α of p21^−/−^ and p21^+/+^ mice at each time point and control. In (**c,e,f**), four mice were analyzed from each group. Two-way analysis of variance and Tukey’s post hoc test for multiple comparisons of paired samples were used. *CI* confidence interval, *CAIA* collagen antibody-induced arthritis, *p21* cyclin-dependent kinase inhibitor 1.

**Figure 2:**
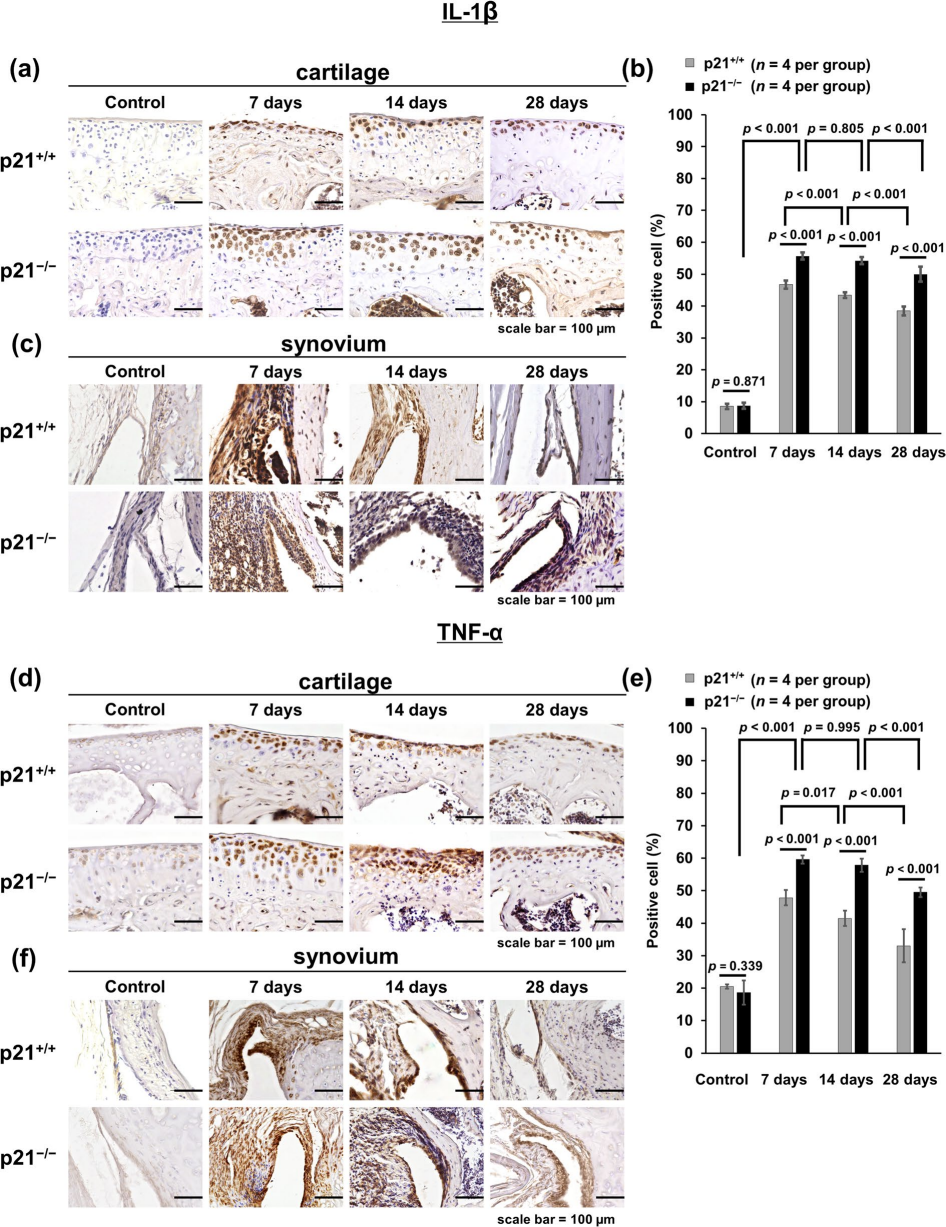
p21 levels influence the number of IL-1β and TNF-α positive cells in the CAIA mouse model. (**a,d**) Cartilage and (**c,f**) synovial tissue samples were collected from the knees of mice after antibody cocktail administration. (**a,c,d,f**) p21^+/+^ mice and p21^−/−^ mice as control, on days 7, 14, and 28. (**b**) The percentage of IL-1β-stained cells (number of positive cells/total number of cells) with 95% CI. The sections were counterstained with hematoxylin. (**e**) The percentage of TNF-α-stained cells (number of positive cells/total number of cells) with 95% CI. In (**b,e**), four mice were analyzed from each group. Two-way analysis of variance and Tukey’s post hoc test for multiple comparisons of paired samples were used. *CI* confidence interval, *CAIA* collagen antibody-induced arthritis, *p21* cyclin-dependent kinase inhibitor 1.

**Figure 3:**
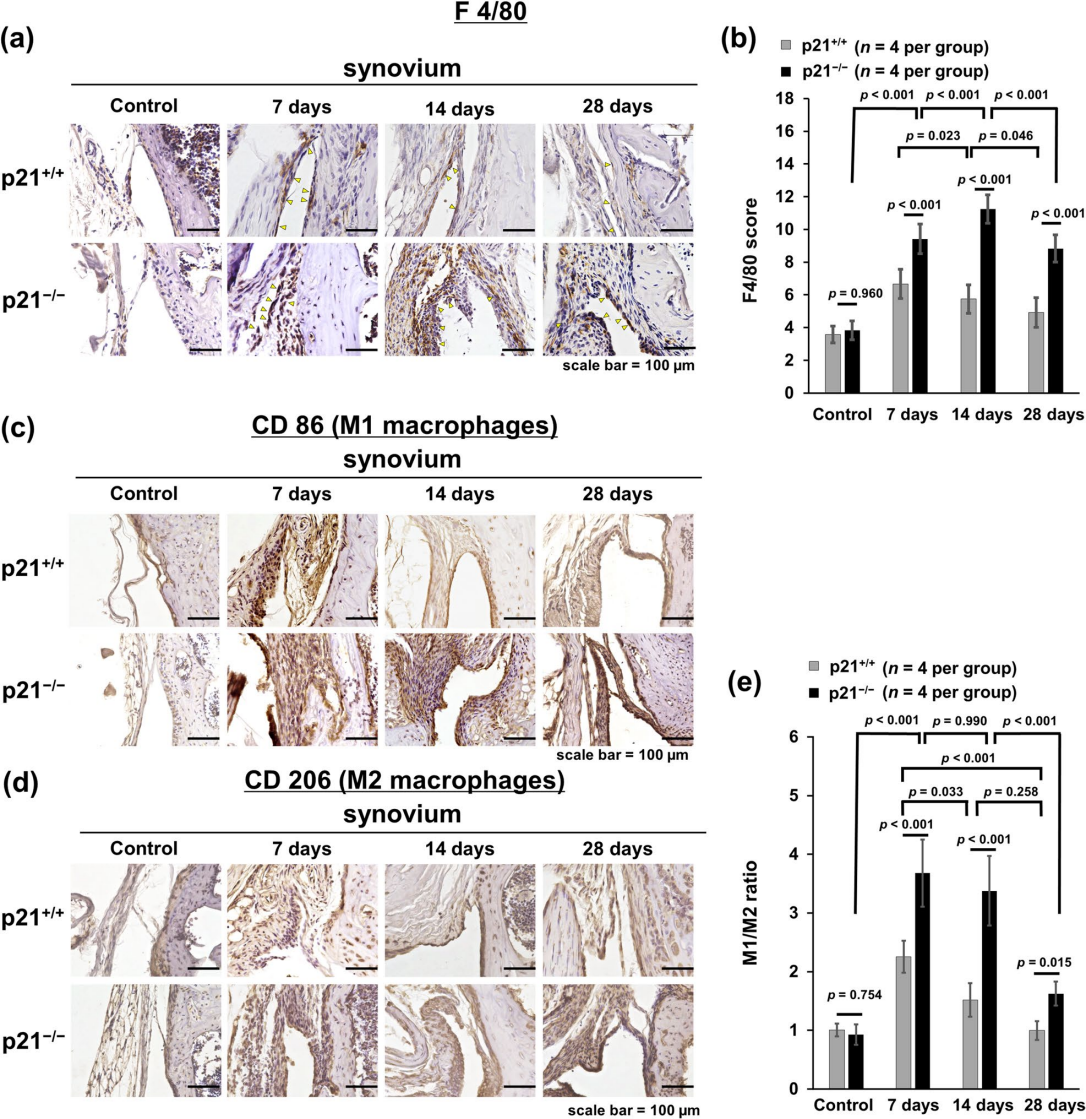
p21 levels influence the number of F4/80- and CD 86-positive cells in the CAIA mouse model. (**a,c,d**) Synovial tissue samples were collected from the knees of mice after antibody cocktail administration. (**a,c,d**) p21^+/+^ mice and p21^−/−^ mice as control, on days 7, 14, and 28. (**b**) Semiquantitative analysis of the infiltration of F4/80-positive cells into the synovial membrane. The average F4/80 score with 95% CI was calculated from all four quadrants. The sections were counterstained with hematoxylin (**e**) The CD86/CD206 expression ratio (M1/M2 ratio) with 95% CI. The sections were counterstained with hematoxylin. In (**b,e**), four mice were analyzed from each group. Two-way analysis of variance and Tukey’s post hoc test for multiple comparisons of paired samples were used. *CI* confidence interval, *CAIA* collagen antibody-induced arthritis, *p21* cyclin-dependent kinase inhibitor 1.

**Figure 4:**
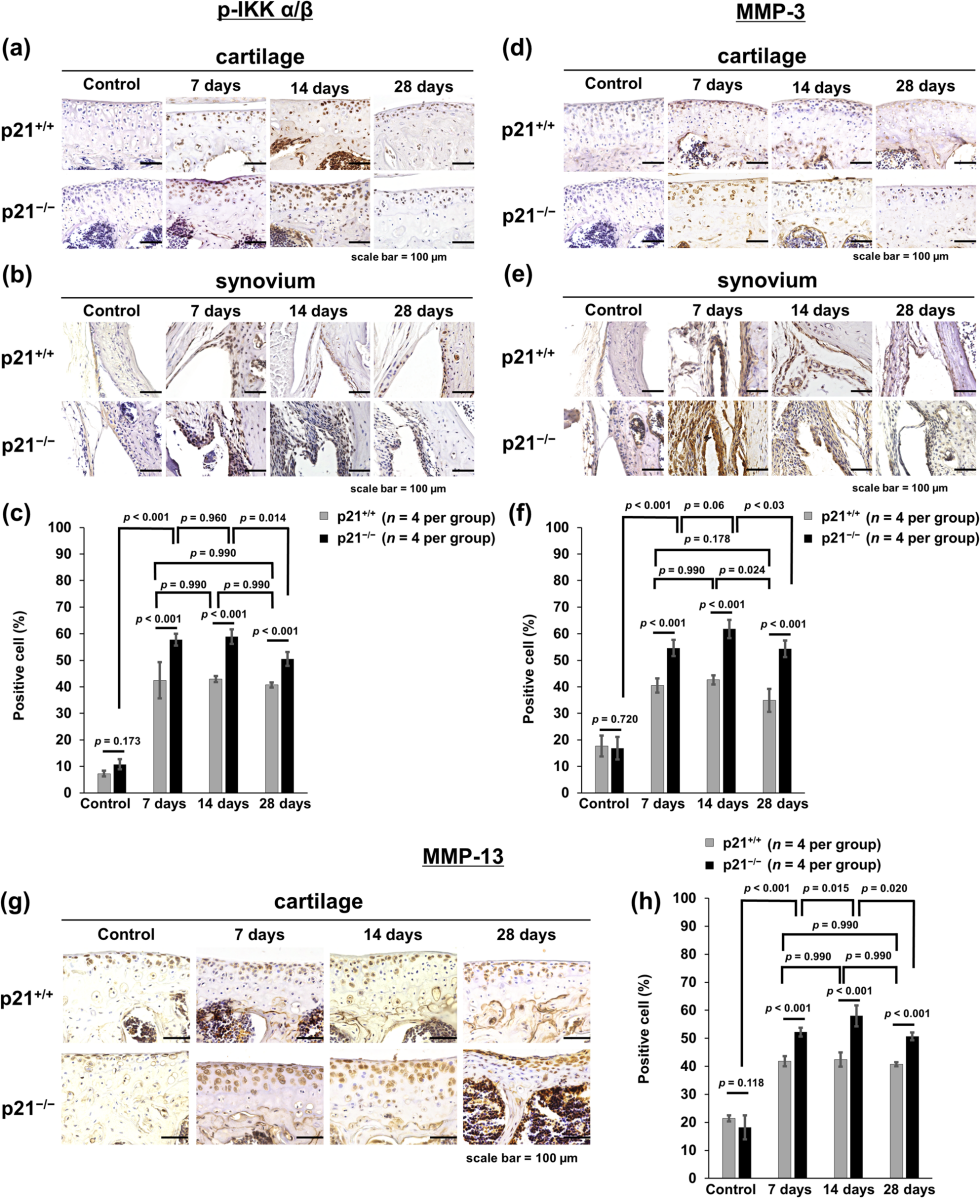
p21 levels influence the numbers of p-IKKα/β-, MMP-3- and MMP-13-positive cells in the CAIA mouse model. (**a,d,g**) Cartilage and (**b,e**) synovial tissue samples were collected from the knees of mice after antibody cocktail administration. (**a,b,d,e,g**) p21^+/+^ mice and p21^−/−^ mice as control, on days 7, 14, and 28. (**c**) The percentage of p-IKKα/β-stained cells (number of positive cells/total number of cells) with 95% CI. (**f**) The percentage of MMP-3-stained cells with 95% CI. (**h**) The percentage of MMP-13-stained cells with 95% CI. The sections were counterstained with hematoxylin. (**c,f,h**), four mice were analyzed from each group. Two-way analysis of variance and Tukey’s post hoc test for multiple comparisons of paired samples were used. *CI* confidence interval, *IKK* IκB kinase complex, *CAIA* collagen antibody-induced arthritis, *p21* cyclin-dependent kinase inhibitor 1.

**Figure 5:**
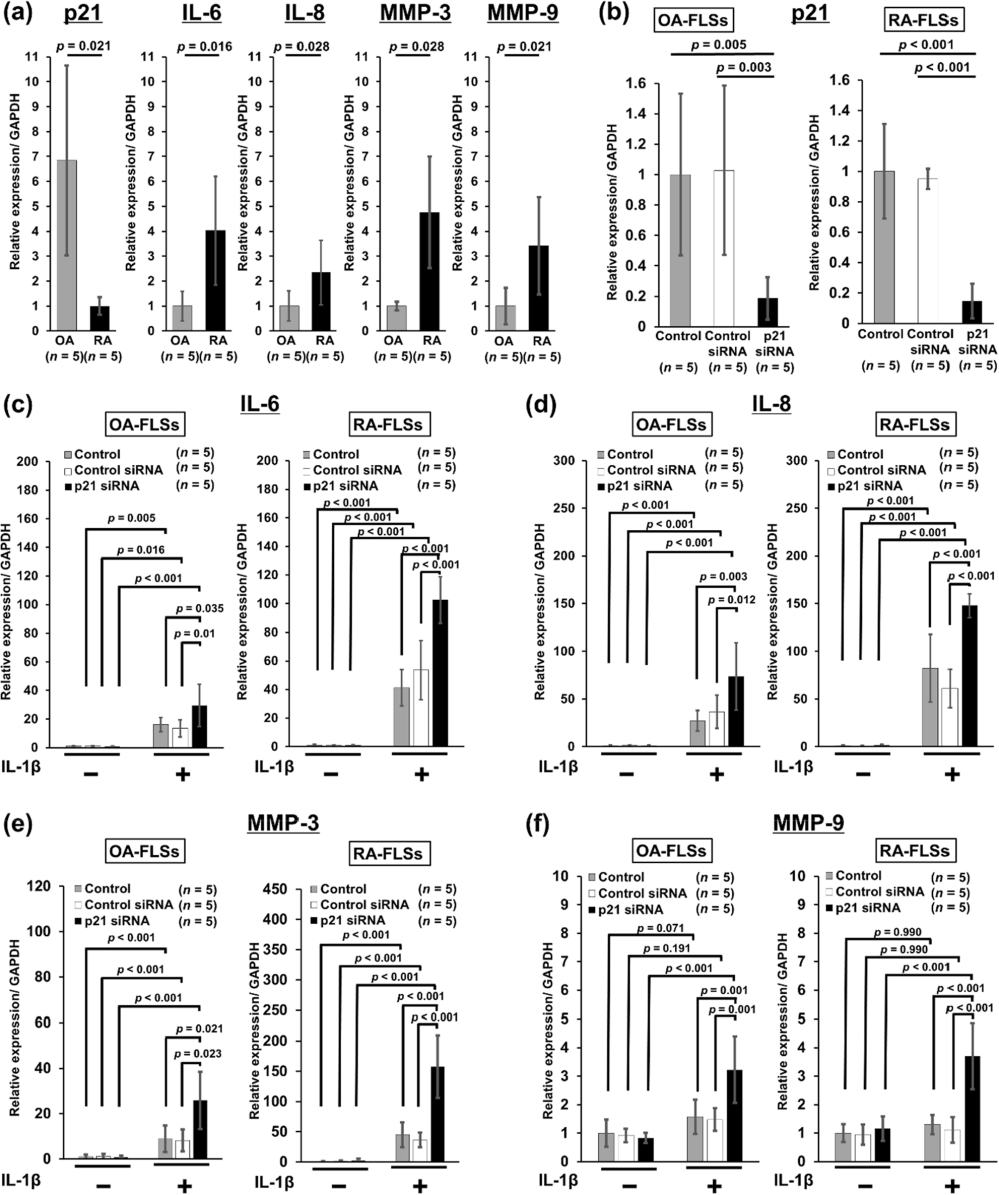
Knockdown of the *p21* gene affects *IL-6*, *IL-8*, and *MMP* expression in rheumatoid arthritis and osteoarthritis synovial tissues. (**a**) The relative expression of *p21*, *IL-6*, *IL-8*, *MMP-3*, and *MMP-9* mRNA was determined in fibroblast-like synoviocytes (FLSs). (**b**) FLSs were transfected with *p21* siRNA or nonspecific control siRNA for 24 h, and the relative expression of *p21* mRNA was determined. The relative expression of (**c**) *IL-6*, (**d**) *IL-8*, (**e**) *MMP-3*, and (**f**) *MMP-9* mRNA was determined after transfection with *p21* siRNA or nonspecific control siRNA and treatment with or without 10 ng/ml recombinant human IL-1β for 24 h. The relative expression of *IL-6*, *IL-8*, *MMP-3,* and *MMP-9* mRNA with respect to the control is shown with 95% CI. Five human synovial tissues were analyzed from each group. In (**a**), the Mann–Whitney U test was used for comparisons between two groups. In (**b**), one-way analysis of variance and Tukey’s post hoc test for multiple comparisons of paired samples were used. In (**c**–**f**), two-way analysis of variance and Tukey’s post hoc test for multiple comparisons of paired samples were used. *CI* confidence interval, *FLSs* fibroblast-like synoviocytes, *p21* cyclin-dependent kinase inhibitor 1.

**Figure 6:**
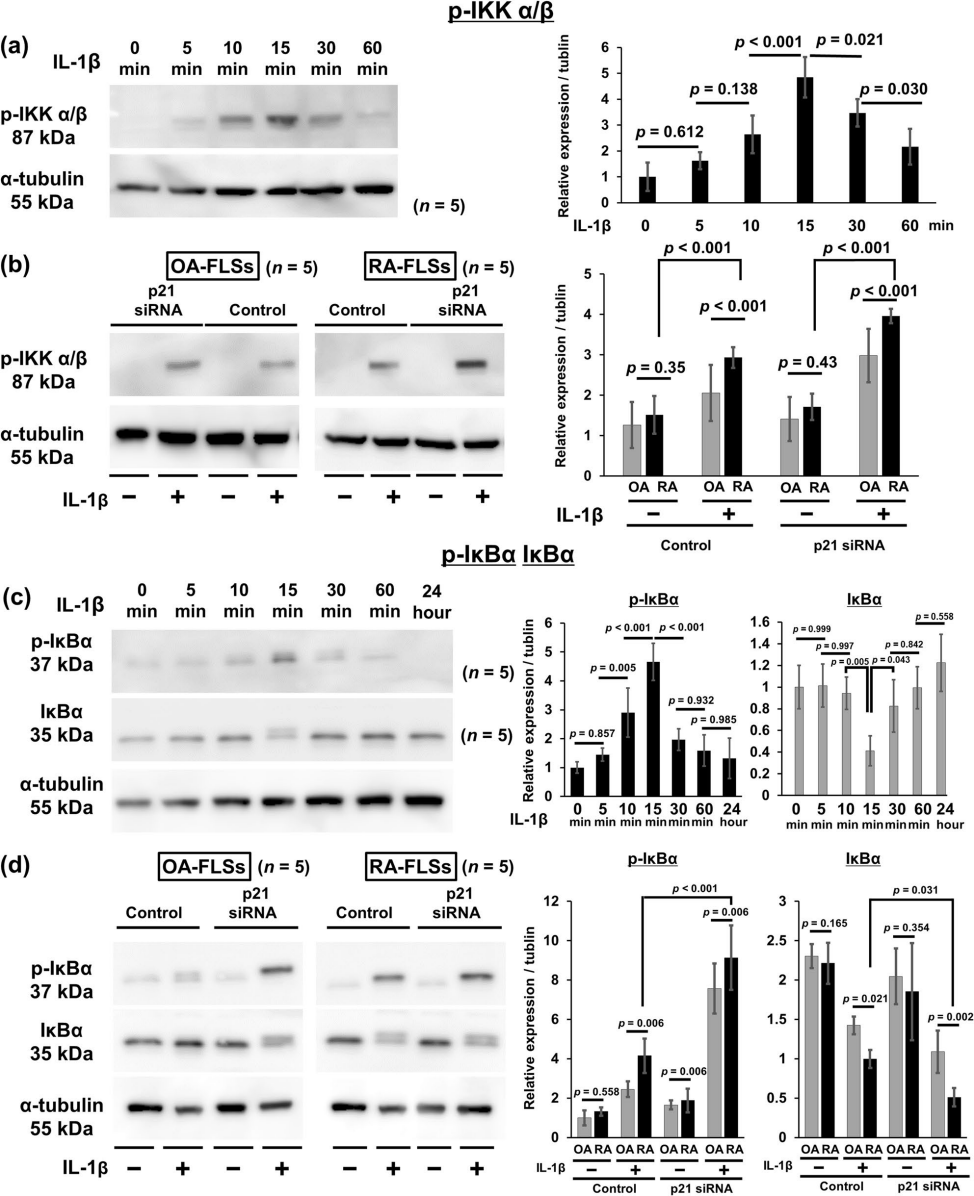
Knockdown of *p21* gene affects IKKα/β and IκBα phosphorylation, and IκBα degradation in rheumatoid arthritis synovial tissues. Western blotting was performed to analyze the (**a**,**c**) time-dependent IL-1β-induced IKKα/β and IκBα phosphorylation, and IκBα degradation. (**b**,**d**) p-IKKα/β, p-IκBα, and IκBα expression in RA and OA synovial cells after IL-1β treatment for 15 min. (**a**–**d**) Expression was determined by semiquantitative analysis of digitally captured images. The samples derive from the same experiment, and that gels/blots were processed in parallel. Full-length blots/gels are presented in Supplementary Fig. S2. Five human synovial tissues were analyzed from each group. In (**a,c**), one-way analysis of variance and Tukey’s post hoc test for multiple comparisons of paired samples were used. In (**b,d**), two-way analysis of variance and Tukey’s post hoc test for multiple comparisons of paired samples were used. *CI* confidence interval, *FLSs* fibroblast-like synoviocytes, *IKK* IκB kinase complex, *IκB* inhibitor of κB, *p21* cyclin-dependent kinase inhibitor 1, *RA* rheumatoid arthritis, *OA* osteoarthritis.

Statistical analysis was performed using one-way (Figs. 5b, 6a,c) or two-way (Figs. 1a,c,e,f, 2b,e, 3b,e, 4c,f,h, 6b,d) analysis of variance and Tukey’s post hoc test for multiple comparisons of paired samples. The Mann–Whitney U test was used to compare two groups in vitro the relative expression of p21, ILs, and MMPs mRNA (Fig. 5a). Results are presented as means with 95% confidence intervals and were considered statistically significant at *P* < 0.05.

### Ethics approval and consent to participate

This study was performed in strict accordance with the recommendations of the Guide for the Care and Use of Laboratory Animals published by the National Institutes of Health (Bethesda, MD, USA). All procedures were approved by the Animal Studies Committee of Kobe University, Japan (permit number: P180404). Synovial tissues were obtained during a total knee joint replacement surgery from five patients with RA and OA. All samples were obtained in accordance with the World Medical Association Declaration of Helsinki Ethical Principles for Medical Research Involving Human Subjects. The study protocol was approved by the Kobe University Graduate School of Medicine Ethics Committee, and all participants provided informed consent.

## Results

### p21-deficient mice were susceptible to joint destruction and severe synovitis and exhibited enhanced systemic inflammatory cytokine expression in vivo

Severe arthritis was observed in p21^−/−^ mice after injection of the monoclonal antibody cocktail. The macroscopic arthritis scores of p21^−/−^ mice were significantly higher than those of p21^+/+^ mice on days 7, 10, 14, and 28 (Fig. 1a). Based on Safranin-O and Fast Green staining, p21^−/−^ mice showed an earlier loss of Safranin-O staining than both the control and p21^+/+^ mice on day 7 (Fig. 1b). Both p21^−/−^ and p21^+/+^ groups showed loss of Safranin-O staining, but the articular surface layer remained intact on day 14 (Fig. 1b). While p21^−/−^ mice showed mid-zone excavation of cartilage tissue, the articular surface of p21^+/+^ mice remained intact on day 28 (Fig. 1b). According to the OARSI cartilage OA-histopathology scoring system, the average sum score of p21^−/−^ mice increased significantly compared with that of p21^+/+^ mice on day 28 (Fig. 1c). Histological analysis using hematoxylin–eosin staining showed that synovitis of the knee joints was more severe in p21^−/−^ mice than in p21^+/+^ mice (Fig. 1d). On days 7 and 14, p21^−/−^ mice showed marked cellular infiltration mixed with lymphoid follicles, multiple-layered synovial lining cells, and villous hyperplasia compared with the control (Fig. 1d). Moreover, on day 28, p21^−/−^ mice displayed severe synovitis, whereas synovitis was attenuated in p21^+/+^ mice at this time point (Fig. 1d). More severe synovitis was observed in p21^−/−^ mice than in p21^+/+^ mice at all time points (Fig. 1d, e). There was no significant difference in synovitis-severity of p21^−/−^ mice between days 7 and 14 (Fig. 1d,e). To analyze p21 involvement in systemic inflammation, we quantified serum levels of IL-1β, IL-6, and TNF-α after the injection of a monoclonal antibody cocktail. p21^−/−^ mice showed higher IL-1β, IL-6, and TNF-α serum levels than p21^+/+^ mice at each time point except for the control (Fig. 1f).

### p21-deficient mice exhibited enhanced local expression of inflammatory cytokine in knee joints

p21^−/−^ mice showed higher IL-1β and TNF-α expression in cartilage tissues than p21^+/+^ mice at each time point except for the control (Fig. 2a,d). IL-1β and TNF-α expression in synovial tissues were also increased in p21^−/−^ mice compared with that in the p21^+/+^ mice at each time point except for the control (Fig. 2c,f). The ratios of IL-1β-positive cells and TNF-α-positive cells in cartilage were significantly higher in p21^−/−^ mice than in p21^+/+^ mice at each time point except for the control (Fig. 2b,e). Of note, those of IL-1β and TNF-α were significantly higher on day 7 and thereafter than those of the control (Fig. 2b,e). There was no significant difference in the positive-cell ratio of p21^−/−^ mice between days 7 and 14 (Fig. 2a,b,d,e).

### p21 deficient mice exhibited enhanced M1 macrophage infiltration in synovial tissues

F4/80-expression levels were elevated in the synovial tissue of p21^−/−^ mice at each time point except for the control, indicating increased macrophage infiltration (Fig. 3a). The F4/80 score was significantly higher in p21^−/−^ mice than in p21^+/+^ mice at each time point except for the control (Fig. 3a,b). CD 86 expression in synovial tissues increased in p21^−/−^ mice compared with p21^+/+^ mice at each time point except for the control (Fig. 3c). CD86/CD206 expression ratio (M1/M2 ratio) was significantly higher in p21^−/−^ mice than in p21^+/+^ mice at each time point except for the control, indicating increased M1 macrophage infiltration (Fig. 3c–e). On day 7 and after that, the F4/80 score and M1/M2 ratio were significantly higher in p21^−/−^ mice than in controls (Fig. 3b,e). There was no significant difference in the M1/M2 ratio of p21^−/−^ mice between days 7 and 14 (Fig. 3e).

### p21-deficient mice increased expression of inflammatory transcription factors and exhibited joint destruction through elevated MMPs expression

p21^−/−^ mice showed higher p-IKKα/β expression in the cartilage and synovial tissues than p21^+/+^ mice at each time point except for the control (Fig. 4a,b). The positive-cell ratio of p-IKKα/β in the cartilage was significantly higher in p21^−/−^ mice than in p21^+/+^ mice at each time point except for control (Fig. 4a,c), and the ratios of p-IKKα/β in p21^−/−^ mice on day 7 and thereafter were significantly higher than those in the control (Fig. 4c). There was no significant difference in the positive-cell ratio of p21^−/−^ mice between days 7 and 14 (Fig. 4c). MMP-3 and MMP-9 expression levels were elevated in the synovial tissues of p21^−/−^ mice at each time point except for the control (Fig. 4e, Supplementary Fig.S1a). The positive-cell ratios of MMP-3 and MMP-13 in the cartilage were significantly higher in p21^−/−^ mice than in p21^+/+^ mice at each time point except for the control (Fig. 4d,f–h), and positive-cell ratios of MMP-3 and MMP-13 were significantly higher on day 7 and thereafter than those of the control (Fig. 4d,f–h).

### Knockdown of *p21* gene expression enhanced *IL-6*, *IL-8* and *MMPs* expression through IL-β stimulation in RA synovial tissues

RT-PCR analysis showed that expression levels of *p21* were lower in human RA FLS than OA FLS and that *IL-6*, *IL-8*, *MMP-3*, and *MMP-9* were highly expressed in RA FLS (Fig. 5a). The knockdown efficacy of p21 inhibited by 85.3% and 81.4% in RA FLS and OA FLS, respectively, after transfection with *p21*-specific siRNA (Fig. 5b). *IL-6* expression significantly increased by 103-fold and 29-fold in *p21* knockdown RA FLS and OA FLS, respectively, in response to IL-β stimulation (Fig. 5c). *IL-8* expression increased significantly by 158-fold and 73-fold in p21 knockdown RA FLS and OA FLS, respectively (Fig. 5f). *MMP-3* expression increased significantly by 157-fold and 26-fold in *p21* knockdown RA FLS and OA FLS, respectively, by IL-β stimulation (Fig. 5e), whereas *MMP-9* expression increased significantly by 3.7-fold and 3.2-fold in *p21* knockdown RA FLS and OA FLS, respectively (Fig. 5f).

### Knockdown of *p21* gene expression enhanced phosphorylation of IKKα/β and IκBα, and degradation of IκBα in RA synovial tissues

Western blot results demonstrated that the expression of p-IKKα/β and p-IκBα markedly increased after 15 min treatment with 10 ng/ml IL-1β (Fig. 6a,c). Therefore, the RA FLS and OA FLS were treated with IL-1β for 15 min in subsequent experiments. The knockdown of *p21* gene expression significantly enhanced IKKα/β phosphorylation in RA FLS compared with OA FLS (Fig. 6b). Western blot also demonstrated that IκBα expression markedly decreased following 15 min treatment with IL-1β (Fig. 6c). The knockdown of *p21* gene expression significantly enhanced the IκBα phosphorylation and IκBα degradation in RA FLS compared with OA FLS (Fig. 6d).

### Inter and intra-rater interclass correlation coefficient

The intra-rater interclass correlation coefficient (ICC) and the inter-rater ICC were calculated to examine the reproducibility of measurements. All measurements were performed twice by one examiner and once by triple-blinded observers. The intra-rater ICCs between the two measurements made by the same examiner for arthritis score (Fig. 1a), OARSI score (Fig. 1c), OARSI-recommended scoring system of hematoxylin (Fig. 1e), positive cell ratio of IL-1β in vivo (Fig. 2b), positive cell ratio of TNF-α in vivo (Fig. 2e), F4/80 score (Fig. 3a), M1/M2 ratio (Fig. 3e), positive cell ratio of p-IKKα/β in vivo (Fig. 4c), positive cell ratio of MMP-3 in vivo (Fig. 4f), positive cell ratio of MMP-13 (Fig. 4h), relative expression of p-IKKα/β in vitro (Fig. 6a,b), and relative expression of p-IκBα and IκBα in vitro (Fig. 6c,d) were 0.988 (95% confidential interval (CI) 0.980–0.994), 0.951 (95% CI 0.891–0.978), 0.843 (95% CI 0.747–0.910), 0.992 (95% CI 0.983–0.996), 0.982 (95% CI 0.965–0.991), 0.928 (95% CI 0.843–0.968), 0.924 (95% CI 0.780–0.983), 0.982 (95% CI 0964–0.991), 0.921 (95% CI 0.845–0.960), 0.970 (95% CI 0.940–0.985), 0.942 (95% CI 0.881–0.969), and 0.899 (95% CI 0.794–0.984), respectively. The inter-rater ICC for arthritis score (Fig. 1a), OARSI score (Fig. 1c), OARSI-recommended scoring system of hematoxylin (Fig. 1e), positive cell ratio of IL-1β in vivo (Fig. 2b), positive cell ratio of TNF-α in vivo (Fig. 2e), F4/80 score (Fig. 3a), M1/M2 ratio (Fig. 3e), positive cell ratio of p-IKKα/β in vivo (Fig. 4c), positive cell ratio of MMP-3 in vivo (Fig. 4f), positive cell ratio of MMP-13 (Fig. 4h), relative expression of p-IKKα/β in vitro (Fig. 6a,b) and relative expression of p-IκBα and IκBα in vitro (Fig. 6c,d) were 0.994 (95% CI 0.990–0.996), 0.928 (95% CI 0.866–0.966), 0.764 (95% CI 0.693–0.874), 0.989 (95% CI 0.981–0.994), 0.987 (95% CI 0.978–0.993), 0.914 (95% CI 0.839–0.959), 0.883 (95% CI 0.761–0.975), 0.984 (95% CI 0971–0.992), 0.949 (95% CI 0.911–0.973), 0.979 (95% CI 0.963–0.989), 0.924 (95% CI 0.854–0.961), and 0.887 (95% CI 0.781–0.979), respectively. These data confirmed experimental reproducibility.

## Discussion

This study demonstrated enhanced cartilage degradation and more severe synovitis in response to systemic inflammation in p21-deficient mice through NF-κB signaling in cartilage and synovial tissues.

Similar to our study, another report has shown increased arthritis scores and observed histological changes, including a marked increase in macrophage infiltration, in the knee synovial membrane of p21^+/+^ CAIA mice^42^. Moreover, we found more severe arthritis phenotype changes in p21^−/−^ mice than in p21^+/+^ mice on day 7 and thereafter, suggesting that *p21* knockdown may exacerbate CAIA and enhance M1 macrophage infiltration in mice. IL-1β expression in p21^−/−^ mice reportedly increased up to 2.4-fold compared with that in p21^+/+^ mice in an experimental endotoxic shock model in vivo, and that in p21^−/−^ bone marrow-derived macrophages with LPS stimulation increased 3.4-folds compared with p21^+/+^ cells in vitro^43^. We have previously reported that *p21* knockout in a murine model of destabilization of the medial meniscus increased IL-1β serum levels and local IL-1β expression in knee joints on days 1 and 56 post-surgery^28^. Macrophages initiate and maintain the inflammation, in the range of antigen presentation and phagocytosis and contribute to immunomodulation via the production of various inflammatory cytokines, including IL-1β and TNF-α^43–45^. The relevance between macrophages and p21 in RA has been highlighted by several studies, including the studies by Trakala et al. that reported increased IL-1β and TNF-α expression in p21^−/−^ macrophages in vitro^46^, and by Mavers et al., who found remarkably increased macrophage infiltration in the ankles of a p21^−/−^ arthritis mouse model and significantly elevated IL-1α serum levels^31^. Our current study also found that p21^−/−^ CAIA model mice exhibited severe joint arthritis with macrophage infiltration, especially M1 macrophages, and elevated IL-1β, IL-6, and TNF-α serum levels, as well as local IL-1β and TNF-α expression in vivo. These findings suggest that activated macrophages enhance systemic and local inflammation in this murine model of RA.

Several reports have shown that IL-1β and TNF-α stimulate NF-κB signaling and induces the expression of MMP-3, MMP-9, and MMP-13^13,47^. It is known that the IKK complex and IκBα proteins play a central role in the regulation of NF-κB activity^48,49^. IκBα protein binds tightly to NF-κB dimer and dampen NF-κB activation. Since bound IκBα requires IKK phosphorylation for basal degradation, increased p-IKKα/β and p-IκBα along with IκBα degradation activate NF-κB. In this study, we confirmed that the expression levels of p-IKKα/β, MMP-3, and MMP-13 in chondrocytes and of p-IKKα/β, MMP-3, and MMP-9 in synovial tissues were elevated in p21^−/−^ mice compared with those in p21^+/+^ mice in vivo. These findings suggest that rapid joint destruction was caused by elevated levels of inflammation induced via p-IKKα/β signaling. Perlman et al. reported that synovial fibroblasts from p21-deficient mice enhance IL-6 and MMP-3 mRNA levels, causing a 100-fold increase in IL-6 protein levels^33^. Hence, alterations in p21 expression may enhance pro-inflammatory cytokine and MMP production, thereby promoting the development of autoimmune diseases^33^. These earlier studies support our results that p21-deficient mice exhibit enhanced MMP-3, MMP-9, and MMP-13 expression through IL-1β- and TNF-α-induced NF-κB signaling.

Consistent with our results, RA FLSs have exhibited decreased p21 expression^33^, higher IL-1β levels^6,50^, higher IL-6 and IL-8 levels^51,52^ compared with OA FLSs. Moreover, other studies have demonstrated increased IL-6 and MMP levels in RA FLSs through TNF-α stimulation^53,54^. Consequently, the decline in p21 expression in RA FLSs might cause severe inflammation through IL-1β-induced NF-κB signaling. Furthermore, we have demonstrated that the knockdown of p21 in FLSs alters the cellular response to IL-1β stimulation. IL-1β stimulation increased IL-6, IL-8, MMP-3, and MMP-9 expression, and enhanced p-IKKα/β, p-IκBα activation, and IκBα degradation in both RA FLSs and OA FLSs. However, the responses to IL-1β in RA FLSs and OA FLSs were different, indicating that decreased expression of p21 in RA joints may account for the observed joint destruction.

This study has a limitation. The applicability of the results from this study to human arthritis is limited using an experimental animal model of antibody to collagen-induced arthritis. The human scenario is much more complex than can be recreated in the CAIA model.

## Conclusions

Overall, we demonstrated that p21-deficient CAIA mice were susceptible to joint cartilage destruction and severe synovitis via IL-1β- and TNF-α-induced inflammation in vivo. We have also shown that knockdown of p21 led to enhanced susceptibility to inflammation through IL-1β stimulation in RA FLSs compared with that in OA FLSs. Therefore, p21 may suppress inflammatory cytokine production, including IL-1β, IL-6, and TNF-α, and represent a potential therapeutic target for novel RA treatment. However, given that p21 is an oncogene involved in cell-cycle regulation, further research is required to verify the viability and safety of p21-based therapies for RA treatment.

## Supplementary Information

Supplementary Information.

## Data Availability

The datasets used and/or analyzed during the current study are available from the corresponding author on reasonable request.

## Abbreviations

RA: Rheumatoid arthritis
IL: Interleukin
TNF-α: Tumor necrosis factor-α
FLSs: Fibroblast-like synoviocytes
MMP: Matrix metalloproteinases
CAIA: Collagen antibody-induced arthritis
p21: Cyclin-dependent kinase inhibitor 1
OA: Osteoarthritis
NF-κB: Nuclear factor kappa-light-chain-enhancer of activated B cells
WT: Wild type
IHC: Immunohistochemistry
IκB: Inhibitor of κB
IKK: Phospho-IκB kinase complex
