# Supplementary material for: Susceptibility of cyclin-dependent kinase inhibitor 1-deficient mice to rheumatoid arthritis arising from interleukin-1β-induced inflammation
Source: Sci Rep. 2021 Jun 15;11:12516. doi: 10.1038/s41598-021-92055-9 (PMC8206139; doi:10.1038/s41598-021-92055-9)
Supplement: Supplementary file 1 — Supplementary Information. [file 41598_2021_92055_MOESM1_ESM.pdf]

Susceptibility of cyclin-dependent kinase inhibitor 1-deficient mice to rheumatoid arthritis arising from interleukin-1β–induced inflammation

Yoshinori Takashima, MD, Shinya Hayashi, MD, PhD, Koji Fukuda, MD, PhD, Toshihisa Maeda, MD, PhD, Masanori Tsubosaka, MD, Tomoyuki Kamenaga, MD, Kenichi Kikuchi, MD, Masahiro Fujita, MD, Yuichi Kuroda, MD, PhD, Shingo Hashimoto, MD, PhD, Naoki Nakano, MD, PhD, Tomoyuki Matsumoto, MD, PhD, Ryosuke Kuroda, MD, PhD

Department of Orthopedic Surgery, Kobe University Graduate School of Medicine, Kobe, Hyogo, Japan

Address correspondence to:

Shinya Hayashi, MD, PhD

Department of Orthopaedic Surgery, Kobe University Graduate School of Medicine, 7-5-2, Kusunoki-chou, Chuo-ku, Kobe 650-0017, Hyogo, Japan; Phone: 81-78-382-5985; Fax: 81-78-351-6944; E-mail: s11793290@yahoo.co.jp

Supplementary Material

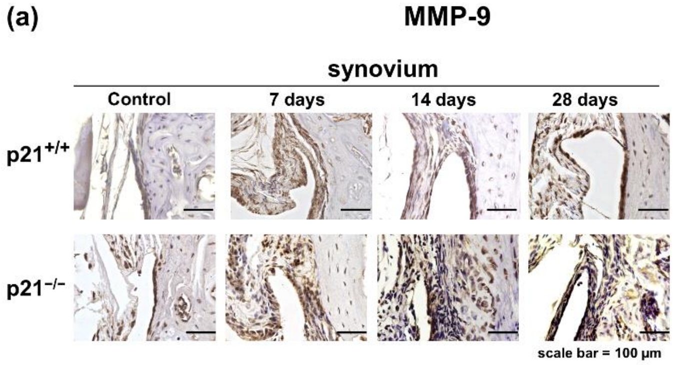

Supplementary Figure S1. p21 levels influence the number of MMP-9-positive cells in the CAIA mouse model. (a) Synovial tissue samples were collected from the knees of mice after antibody cocktail administration. (a) $p21^{+/+}$ mice and $p21^{-/-}$ mice as controls and on days 7, 14, and 28. The sections were counterstained with hematoxylin. Four mice were analyzed for each group. CAIA: collagen antibody-induced arthritis; p21: cyclin-dependent kinase inhibitor 1; MMP, matrix metalloproteinase.

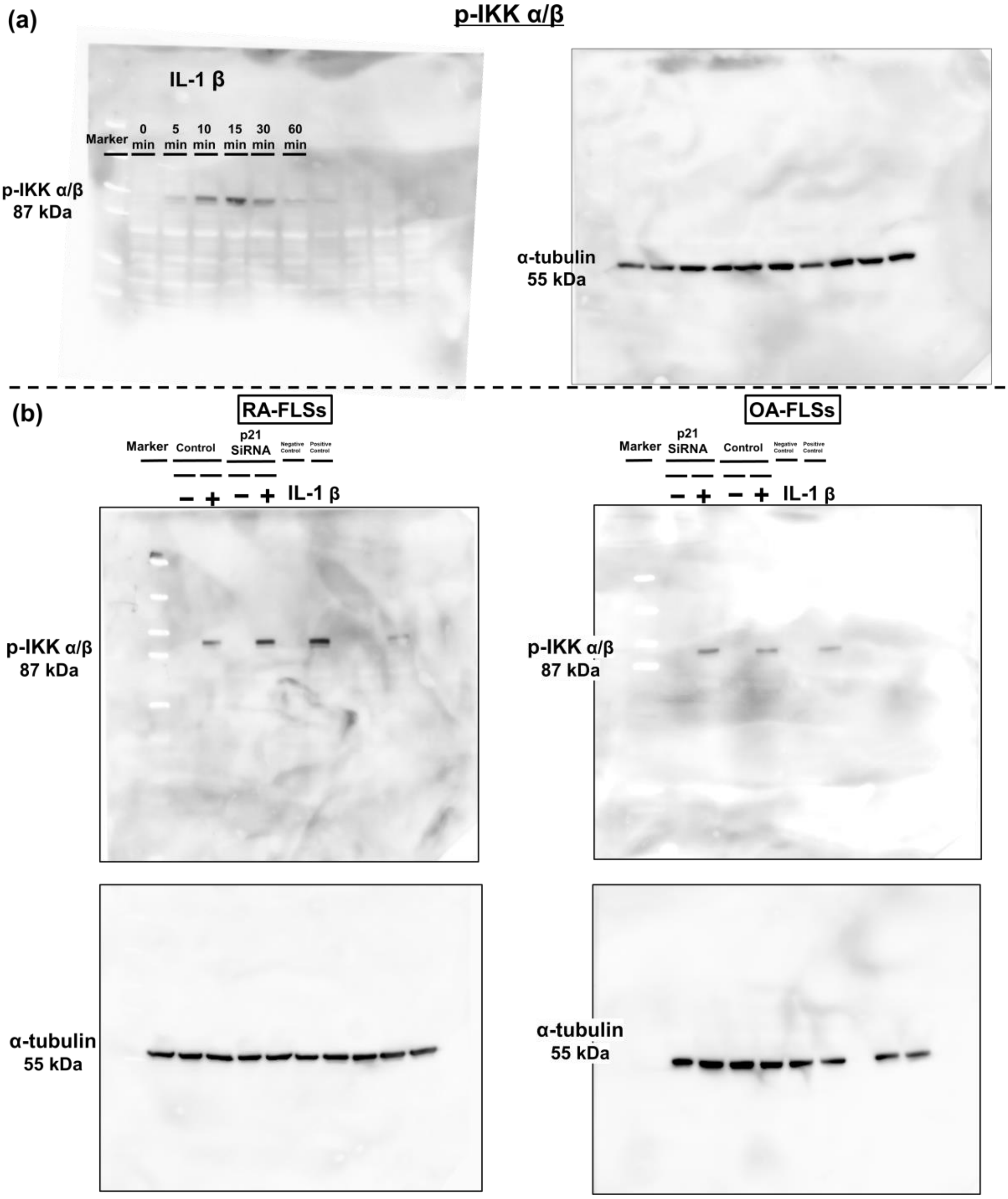
(a)
p-IKK α/β
IL-1 β
Marker
0 min
5 min
10 min
15 min
30 min
60 min
p-IKK α/β
87 kDa
α-tubulin
55 kDa
(b)
RA-FLSs
Marker
Control
p21 SiRNA
Negative Control
Positive Control
− + − + IL-1 β
p-IKK α/β
87 kDa
α-tubulin
55 kDa
OA-FLSs
Marker
p21 SiRNA
Control
Negative Control
Positive Control
− + − + IL-1 β
p-IKK α/β
87 kDa
α-tubulin
55 kDa

(c)

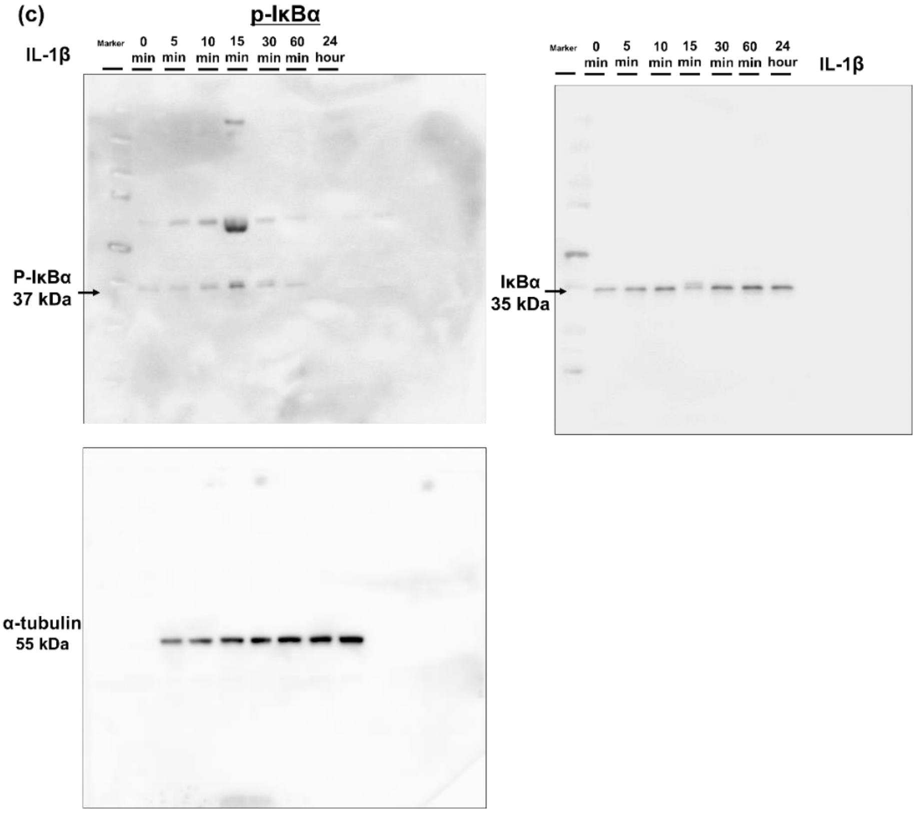

p-IκBα
IL-1β
Marker
0 min
5 min
10 min
15 min
30 min
60 min
24 hour
P-IκBα
37 kDa
α-tubulin
55 kDa
Marker
0 min
5 min
10 min
15 min
30 min
60 min
24 hour
IL-1β
IκBα
35 kDa

(d)

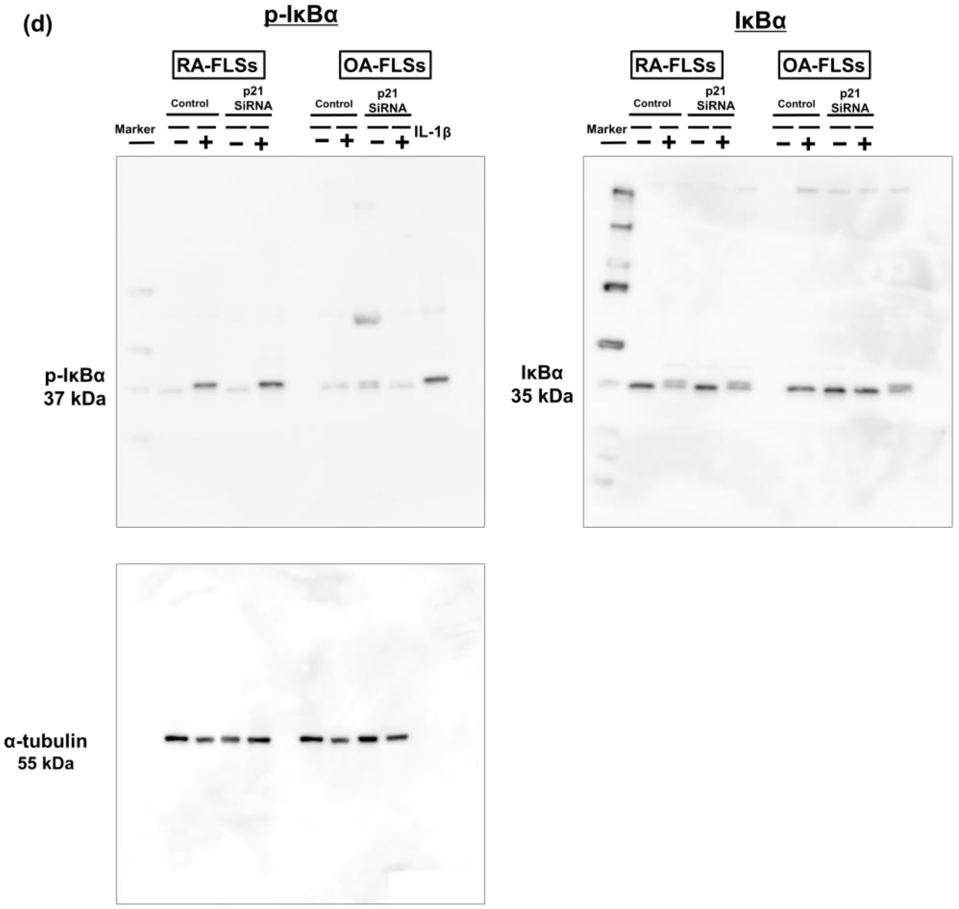

p-IκBα
RA-FLSs
OA-FLSs
Control
p21 SiRNA
Control
p21 SiRNA
Marker
− + − + − + − +
IL-1β
p-IκBα
37 kDa
α-tubulin
55 kDa
IκBα
RA-FLSs
OA-FLSs
Control
p21 SiRNA
Control
p21 SiRNA
Marker
− + − + − + − +
IκBα
35 kDa

Supplementary Figure S2. Full-length blots/gels of (a) time-dependent IL-1β-induced phosphorylation of IKKα/β, and (b) p-IKKα/β expression in RA and OA synovial cells after IL-1β treatment for 15 min. Full-length blots/gels of (c) time-dependent IL-1β-induced phosphorylation of IκBα, and (d) p-IκBα and IκBα expression in RA and OA synovial cells after treatment with IL-1β for 15 min. (a, b, c, d) Expression was determined by semiquantitative analysis of digitally captured images. The samples derive from the same experiment, and that gels/blots were processed in parallel. CI: confidence interval; IKK: IκB kinase complex; IκB: inhibitor of κB, p21: cyclin-dependent kinase inhibitor 1; RA: rheumatoid arthritis; OA: osteoarthritis.

Supplementary Table S1. Primers used for a quantitative reverse transcriptase-polymerase chain reaction.

	Primer sequence (5'-3’)	
Gene	Forward	Reverse
GAPDH	*GTTCGACAGTCAGCCGCATC*	*GGAATTTGCATGGGTGGA*
p21	*GCGATGGAACTTCGCTTTGT*	*GGGCTTCCTCTTGGAGAAGAT*
IL-6	*AGACAGCCACTCACCTCTTCAG*	*TTCTGCCAGTGCCTCTTTGCTG*
IL-8	*GAGAGTGATTGAGAGTGGACCAC*	*CACAACCCTCTGCACCCAGTTT*
MMP3	*ATTCCATGGAGCCAGGCTTTC*	*CATTTGGGTCAAACTCCAACTGTG*
MMP9	*TGTAAAACGACGGCCAGT*	*CAGGAAACAGCTATGACC*

GAPDH, Glyceraldehyde 3-phosphate dehydrogenase

p21, cyclin-dependent kinase inhibitor 1

IL, Interleukin

MMP, matrix metalloproteinase
